# Exogenous Salicylic Acid Improves Fruit Yield and Quality of *Lycium barbarum* Under Summer High Temperature

**DOI:** 10.3390/cimb48080836

**Published:** 2026-08-17

**Authors:** Xiaoya Qin, Qi Li, Yue Yin, Yunfang Fan, Xiaojie Liang, Ken Qin

**Affiliations:** 1 Wolfberry Science Institute, Ningxia Academy of Agriculture and Forestry Sciences, Yinchuan 750002, China; qinxiaoya@whu.edu.cn (X.Q.);; 2 National Wolfberry Engineering Research Center, Yinchuan 750002, China; 3 State Key Laboratory for Quality Ensurance and Sustainable Use of Dao-Di Herbs, Institute of Chinese Materia Medica, China Academy of Chinese Medical Sciences, Beijing 100700, China

**Keywords:** goji berry, salicylic acid, summer high temperature, fruit yield, fruit quality

## Abstract

Global warming poses an escalating threat to crop yield and quality, particularly for thermosensitive medicinal-edible plants such as goji berry (*Lycium barbarum*). Although salicylic acid (SA) is recognized for enhancing plant thermotolerance, its effects on fruit productivity and quality under summer high temperatures remain inadequately quantified. This study investigated the physiological, metabolic, and transcriptomic responses of field-grown Ningqi No. 7 (N7) goji berry to exogenous SA application under summer heat stress. SA treatment significantly increased fruit yield by 22%, while reducing leaf defoliation rate by 32%. Metabolite profiling revealed that SA elevated total soluble sugar content by 30% and flavonoid concentration by 14% but decreased total organic acid content by 16%. Transcriptomic analysis identified SA-induced differential expression of genes involved in sugar metabolism (phosphofructokinase), secondary biosynthesis (flavonoid and alkaloid related pathways), and stress responses (HSPs, E3 ubiquitin ligases, SRK2). These results demonstrate that exogenous SA enhances goji berry productivity and improves fruit taste (via increased sugar/organic acid ratio) and functional quality under summer high temperatures. Consequently, SA application thus represents a promising and cost-effective strategy for climate-resilient goji berry cultivation.

## 1. Introduction

Goji berry (*Lycium barbarum* L.), a deciduous shrub belonging to the Solanaceae family, is globally distributed with primary cultivation concentrated in the Ningxia, Qinghai, and Gansu provinces of China. The genus comprises approximately 80 species, among which *L. barbarum* and *L. chinense* are the most economically significant [1,2]. Goji berry is unique as a dual-purpose medicinal and food plant; its fruits have long been recognized in traditional Chinese medicine for their immunostimulatory, anti-aging, and hepatoprotective properties. In recent years, global demand for goji berry has risen sharply, driven by increasing consumer awareness of its functional health benefits, including high levels of carotenoids, flavonoids, and polysaccharides [3,4,5]. However, the sustainable production of goji berry is increasingly threatened by environmental constraints. With the intensification of the greenhouse effect, the global climate is undergoing warming, characterized by rising temperatures. The mean global temperature increased by 0.6 °C during the 20th century [6]. Projections indicate that global warming in the 21st century will exceed 1.5 °C to 2 °C unless substantial reductions in carbon dioxide and other greenhouse gases are achieved in the coming decades. This results in severe economic yield losses and significantly impacts agricultural systems [7]. Among these, summer heat waves have emerged as a primary limitation, causing substantial declines in fruit yield, deterioration of quality attributes, and increased susceptibility to diseases and pests, thereby severely hindering the development of the goji berry industry [8]. Understanding the physiological basis of heat-induced damage and developing effective mitigation strategies have therefore become urgent priorities for goji berry production under climate change.

High temperature constitutes a primary environmental stress for crops throughout their life cycle, from seed germination to reproductive maturity. Unlike animals, plants are sessile organisms that cannot escape adverse conditions; instead, they perceive ambient temperature and relay these signals to regulate physiological and developmental responses through a complex gene expression network [9,10]. At the cellular level, heat stress induces protein denaturation, inactivation of chloroplastic enzymes, increased fluidity of membrane lipids, and arrest of photosystem function [11]. These primary injuries trigger secondary oxidative damage through excessive accumulation of reactive oxygen species (ROS), ultimately compromising cellular integrity and organ function.

Plants exhibit greater sensitivity to heat stress during reproductive stages compared to vegetative growth [12,13]. The growth and development of floral organs are closely linked to yield and quality outcomes. High-temperature stress impairs flower bud differentiation, leading to floral organ malformation [13,14], and adversely affects pollen formation by reducing pollen quantity and viability, with the magnitude of impact increasing with temperature intensity [15,16,17,18]. The abnormal development of floral organs, reduced pollen activity, and flower abortion directly lower seed setting or fruit setting rates, thereby reducing final yield. Furthermore, high temperature accelerates plant developmental processes and shortens fruit maturation periods, resulting in abnormal fruit growth and compromised quality attributes, including reduced sugar accumulation, altered organic acid profiles, and diminished secondary metabolite content [19,20,21].

For goji berry specifically, the reproductive phase coincides with summer months when ambient temperatures frequently exceed optimal ranges. Heat stress during flowering and early fruit development has been associated with increased flower drop, reduced fruit set, and diminished accumulation of pharmacologically active compounds such as flavonoids and polysaccharides [8]. However, the precise physiological and molecular mechanisms by which high temperature affects goji berry fruit development and quality remain inadequately characterized, highlighting the need for targeted investigations in this crop.

SA is a simple phenolic compound first identified in 1979 for its role in plant defense against pathogens [22,23]. Beyond its well-established function in systemic acquired resistance (SAR), SA functions as a key signaling molecule that mediates plant responses to various abiotic stresses, including extreme temperatures, salinity, and drought [24,25,26]. Exogenous SA application has been shown to significantly elevate endogenous SA levels, thereby modulating plant defense mechanisms and stress adaptation [27,28].

The protective role of SA against heat stress has been demonstrated across multiple crop species, including cucumber, tobacco, mustard, and grape [29]. Mechanistically, SA enhances plant thermotolerance through multiple pathways: (1) it stimulates antioxidant enzyme activities and reduces ROS accumulation, thereby mitigating oxidative damage [30,31]; (2) it promotes the accumulation of heat shock proteins (HSPs) that function as molecular chaperones to maintain protein homeostasis under thermal stress [32]; (3) it regulates Ca^2+^ homeostasis and lipid peroxidation; and (4) it engages in crosstalk with other phytohormone signaling pathways to coordinate stress responses [33,34].

Importantly, SA exhibits a dose-dependent influence, where varying concentrations lead to distinct physiological responses within the same plant species. Lower concentrations typically promote growth and stress adaptation, while higher concentrations may induce phytotoxicity or adverse effects [27,28]. This concentration-dependent duality necessitates careful optimization of SA application protocols for specific crops and stress conditions.

Despite extensive research on SA-mediated thermotolerance in model and major crop species, its effects on fruit quality parameters-particularly the balance between primary metabolites (sugars, organic acids) and secondary metabolites (flavonoids, alkaloids) remain poorly understood. Furthermore, the molecular mechanisms by which SA reprograms metabolic pathways under heat stress have not been systematically investigated in medicinal-edible plants such as goji berry.

Given the rising global temperatures and the economic importance of goji berry, there is an urgent need to develop effective, low-cost strategies to sustain fruit production and quality under heat stress. SA represents a promising candidate due to its established role in stress mitigation and its affordability for field application. However, the specific effects of SA on goji berry yield and quality under realistic summer heat conditions, and the underlying molecular mechanisms, remain largely unexplored.

We therefore hypothesized that exogenous SA application enhances goji berry thermotolerance by reprogramming primary and secondary metabolic pathways, leading to improved fruit yield and quality attributes under high-temperature stress. To test this hypothesis, the present study was designed with the following specific objectives: To evaluate the effects of exogenous SA application on agronomic traits, including fruit yield, fruit size, and leaf defoliation rate, in field-grown N7 goji berry under summer heat stress; to quantify SA-induced changes in primary metabolites (soluble sugars and organic acids) and secondary metabolites (flavonoids and alkaloids) that determine fruit taste and functional quality; and to elucidate the molecular mechanisms underlying these metabolic changes through transcriptomic analysis of SA responsive genes and pathways.

The findings of this study are expected to provide a scientific basis for the practical application of SA in goji berry cultivation and to contribute to the broader understanding of SA-mediated stress tolerance in medicinal-edible plants under climate change scenarios.

## 2. Materials and Methods

### 2.1. Plant Material and Experimental Design

In this study, we selected the dominant varieties of goji berry, *Lycium barbarum* Ningqi No. 7 (N7) as the experimental material, due to its large fruit size, favorable fruit coloration and quality, ease of drying, and self-cross affinity advantages, which was developed by Qin Ken in 2010. The N7 seeds were used in the germination experiment, and the N7 fruits were collected and used in the field experiment.

In the germination experiment, the seeds of N7 were placed in Petri dishes lined with moist filter paper impregnated with sterile water or SA solutions at concentrations of 25 μM, 50 μM, and 100 μM, respectively. These dishes were subsequently transferred to an artificial climate chamber maintained at a constant temperature of 35 °C, light intensity is 50 µmol·m^−2^·s^−1^, and the relative humidity is 70%, with a photoperiod of 10 h light and 14 h darkness. Seed germination rate was recorded daily.

In the field experiment, the fruit samples were obtained from N7 plants. The experiment was performed in Bairuiyuan Goji Co., Ltd., Zhongning Goji Planting Base (located in Zhongning County, Zhongwei, China, coordinates approximately 37.5° N, 105.7° E). The research consisted of a control group (sprayed with leaf fertilizer) and an experiment group (sprayed with leaf fertilizer and SA). The leaf fertilizer contained potassium dihydrogen phosphate (300 g/acre), magnesium sulfate (100 g/acre), molybdenum ammonium sulfate (10 g/acre), amino acid leaf fertilizer concentrate (0.2 L/acre). The concentration of SA used was 50 g/acre. A total of 100 L water was used per acre of land. The foliar spray was applied during the peak fruit maturity stage of goji berries (from late June to early July), with an interval of approximately 7 days between successive applications.

Yield statistics and biochemical assays: only red-ripe fruits were harvested for yield calculation and edible quality assessment, so only these red-ripe fruits were collected as samples for further study, in which samples from control group were denoted as CK, and samples from experimental group were denoted as SA.

Transcriptome and metabolome analysis: to simultaneously evaluate the effects of SA on both fruit quality (assessed in red-ripe fruits) and fruit development (assessed in immature green fruits), we collected and analyzed both red and green fruits from each treatment group. Four group samples were designated as NX- red, NX- red-SA, NX-green, and NX-green-SA. The NX-red-SA and NX-green-SA samples correspond to red and green fruits, respectively, which were harvested seven days after treatment with SA. In contrast, the NX-red and NX-green samples represent the untreated controls, consisting of red and green fruits collected at the same developmental stage without SA application.

### 2.2. Yield Statistics

Fallen leaves measurement: During July, a collection cloth was placed under ten representative plants per treatment. Fallen leaves were collected daily throughout the entire month of July. The total number of fallen leaves was recorded, and the average leaf drop per plant was calculated for each treatment. Each treatment consisted of three replicate groups (ten plants per replicate), and the mean value was derived from these three replicates.

Sample collection and yield trait measurements: Fruits were harvested when they were fully red and approximately 90% ripe. For each treatment, five representative plants were selected using the five-point sampling method, and all fruits on each selected plant were completely picked. All the fruits were weighed and dried, a small quantity of fruits were rapidly frozen in liquid nitrogen and then stored in a −80 °C refrigerator for subsequent experiments such as component and transcriptome and metabolome analysis.

Fresh and dry fruit weight: Immediately after harvest, the total fresh fruit weight per plant was measured using an electronic balance with 0.01 g precision. The fresh fruits were then placed in a forced-air drying oven and dried at 65 °C until constant weight (approximately 72 h). After cooling to room temperature, the dry fruit weight was determined using the same balance.

Seed number and weight: 10 dried fruits were randomly taken from each treatment, and the pericarps were manually removed to count the number of fully developed seeds. The seeds were then dried in an oven at 65 °C and weighed using an electronic balance with 0.001 g precision. Additionally, one gram of seeds was weighed and the number were counted. One thousand seeds were also counted and weighed. Every measurement was repeated three times.

### 2.3. Flavonoid Content Measurement

The total flavonoid content was determined by the NaNO_2_-Al(NO_3_)_3_ colorimetric method using rutin as the standard, with absorbance measured at 510 nm, following the protocol described by Mi et al. for *Lycium barbarum* [35]. Freshly collected samples were dried at 80 °C until a constant weight was achieved, followed by grinding. The resulting powder was sieved through a 40-mesh screen. Approximately 0.05 g of the sieved material was accurately weighed, mixed with 1 mL of extraction solvent, and vortexed at 60 °C for 2 h. Subsequently, the mixture was centrifuged at 25 °C for 10 min, and the supernatant was collected for analysis. Absorbance was measured at 510 nm. The flavonoid content was calculated using the following formula:

Flavonoid content (mg/g dry weight, DW) = (ΔA − 0.0007) ÷ 5.02 ÷ (W/V_t_) = 0.199 × (ΔA − 0.0007) ÷ W

ΔA = measured absorbance;
V_t_ = total volume of extraction liquid added (1 mL);
W = sample mass (g).

### 2.4. Organic Acid Content Measurement

Total acid content was determined by the acid–base titration method according to the national standard GB 12456-2021 (National Food Safety Standard-Determination of Total Acid in Foods). Based on the principle of acid–base neutralization, titration was performed using a standardized sodium hydroxide solution. A 1 g sample was homogenized with 10 mL of distilled water, followed by centrifugation at 8000 *g* and 4 °C for 10 min. The supernatant was collected, and a 2 mL aliquot was transferred to a small beaker containing 8 mL of distilled water. Subsequently, 20 μL of reagent 1 was added, and the mixture was titrated with reagent 2 until a persistent light red endpoint was achieved. The volume of NaOH consumed was recorded. (Reagent 1: 1% phenolphthalein, 1 g phenolphthalein is dissolved in 60 mL anhydrous ethanol and 40 mL water; Reagent 2: 0.01 mol/L NaOH, 0.04 g NaOH dissolved in 100 mL water). Acid content % (citric acid) = V × 10^−3^ × C_NaOH_ (mol/L) × K × V_extract_/V_sample_ ×100/W = 1.28 × V/W. C_NaOH_: 0.01; K (molar mass conversion coefficient): 64; V_extract_: 10 mL; V: Consumption of sodium hydroxide volume; W: Sample quality, g.

### 2.5. Sugar Content Measurement

Soluble sugar content was determined using the anthrone-sulfuric acid colorimetric method as described previously. The sample was dried, pulverized, and weighed to an approximate mass of 0.02 g. It was then combined with 2 mL of extraction solvent and thoroughly homogenized. The homogenate was transferred to a centrifuge tube and placed in an oven at 90 °C, where it was hydrolyzed for 3 h while sealed to minimize solvent evaporation. After hydrolysis, the tube was cooled and centrifuged at 10,000 *g* for 10 min. The supernatant was diluted 10-fold by mixing 100 μL supernatant with 900 μL distilled water. The diluted mixture was incubated in a 90 °C water bath for 20 min and subsequently cooled under running water. Finally, 1 mL of the prepared solution was transferred to a 1 mL glass colorimetric dish, and absorbance was measured at 490 nm.

Calculate ∆A = A_determination_ − A_blank_.

Total sugar content (mg/g) = (∆A − 0.0181) ÷ 13.689 × V_extract_ × Dilution ratio ÷ W = 1.46 × (∆A − 0.0181) ÷ W. V_extract_: Volume of the extract, 2 mL; Dilution ratio: 10; W: Sample quality, g.

### 2.6. Transcriptome Detection Analysis

For library construction, total RNA exceeding 1 μg was used as the starting material. Polyadenylated mRNA was enriched using oligo (dT) magnetic beads, followed by random fragmentation in NEB fragmentation buffer via divalent cations. First-strand cDNA synthesis was performed using the reverse transcriptase system, with fragmented mRNA as template and random oligonucleotides as primers. The RNA strand was subsequently degraded by RNase, and second-strand cDNA was synthesized using DNA polymerase I with dNTPs as substrates. The purified double-stranded cDNA underwent end repair, A-tailing, and adapter ligation. cDNA fragments of approximately 200 bp were selected using AMPure XP beads, amplified by PCR, and purified again with AMPure XP beads to generate the final library.

Following library preparation, initial quantification was performed using a Qubit 2.0 fluorometer (Invitrogen, Thermo Fisher Scientific, Carlsbad, CA, USA), and libraries were diluted to 1.5 ng/μL. Insert size distribution was assessed with an Agilent 2100 bioanalyzer (Agilent Technologies, Waldbronn, Germany), and the effective library concentration (exceeding 2 nM) was accurately determined by qRT-PCR to ensure quality.

After passing quality control, libraries were pooled based on effective concentration and desired sequencing depth for Illumina sequencing. The raw sequencing data were subjected to quality filtering using the software fastp v0.19.3. The filtering criteria included: (1) removal of adapter-containing reads; (2) elimination of paired-end reads if any read contained over 10% of bases classified as “*N*”; (3) removal of paired-end reads if the proportion of low-quality bases (Phred quality score ≤ 20) within any read exceeded 50% of its total bases. All downstream analyses were performed using the resulting high-quality clean reads.

The reference genome sequence and its corresponding annotation file were retrieved from the specified public database. Genome indexing was carried out with HISAT2, followed by alignment of the clean reads to the reference genome. Novel gene prediction was conducted using StringTie, which employs a network flow algorithm combined with optional de novo assembly to reconstruct transcript isoforms. Compared to other tools such as cufflinks, stringtie demonstrates superior performance in generating more complete and accurate transcripts with enhanced computational efficiency.

Subsequently, gene-level read counts were quantified using feature counts. Based on the gene lengths, FPKM (fragments per kilobase of transcript per million mapped reads) values were calculated for each gene. FPKM represents a widely adopted metric for estimating gene expression levels in RNA-seq studies. Differential expression analysis between groups was conducted with DESeq2 v1.22.1, and *p*-values were adjusted using the Benjamini–Hochberg method. Differentially expressed genes (DEGs) were identified using the criteria of |log_2_(fold change)| ≥ 1 and adjusted *p*-value < 0.05.

### 2.7. Metabolome Assay Analysis

The goji berry fruit samples designated for analysis were subjected to vacuum freeze-drying using a lyophilizer (Scientz-100 F, Ningbo Scientz Biotechnology Co., Ltd., Ningbo, China). Subsequently, the samples were pulverized into a fine powder employing a grinder (MM 400, Retsch, Haan, Germany) at a frequency of 30 Hz for 1.5 min. Precisely 100 mg of the resulting powder was weighed and extracted with 1.2 mL of 70% methanol. The mixture was vortexed for 30 s at 30 min intervals, repeating this procedure six times in total. Following this, the samples were stored overnight at 4 °C for refrigeration. After centrifugation at 13,800 *g* for 10 min, the supernatant was carefully collected and filtered through a 0.22 μm microporous membrane. The filtrate was then transferred to injection vials for subsequent ultra-performance liquid chromatography-tandem mass spectrometry (UPLC-MS/MS) analysis.

Chromatographic separation was conducted using an ExionLC™ AD system equipped with an Agilent SB-C18 column (1.8 µm, 2.1 mm × 100 mm). The mobile phase comprised 0.1% formic acid in water (solvent A) and acetonitrile (solvent B). A gradient elution program was employed as follows: 0–9 min, 5–95% B; 9–10 min, 95% B; 10–11 min, 95–5% B; 11–14 min, 5% B. The flow rate was maintained at 0.35 mL/min, the column temperature was set at 40 °C, and the injection volume was 2 μL. Mass spectrometry (MS) analysis was performed utilizing a system equipped with an electrospray ionization (ESI) source. The ion source parameters were configured as follows: source temperature, 500 °C; ion spray voltage (IS), set to +5500 V in positive mode and −4500 V in negative mode; ion source gas I (GSI), gas II (GSII), and curtain gas (CUR) were maintained at 50, 60, and 25 psi, respectively. Collision-induced dissociation (CID) energy was established at a high level. Scanning was conducted using a triple quadrupole (QQQ) instrument in multiple reaction monitoring (MRM) mode, with nitrogen employed as the collision gas at a medium setting. For each MRM transition, the declustering potential (DP) and collision energy (CE) were individually optimized through meticulous tuning of the declustering voltage and collision energy. In the course of data processing, redundant signals, encompassing isotopic peaks, and multiply charged ions, were systematically eliminated.

The analytical instrumentation primarily comprised an ultra-high-performance liquid chromatography system (https://www.shimadzu.com.cn/, accessed 23 July 2026) coupled with a tandem mass spectrometer (Applied Biosystems 4500 QTRAP, Applied Biosystems, Foster City, CA, USA). Mass spectrometric data acquisition and processing were conducted using the Analyst software (version 1.6.3).

### 2.8. Statistical Analysis

All statistical analyses and graphical visualizations were performed using Origin 2021b (OriginLab Corporation, Northampton, MA, USA) and GraphPad Prism (version 10.4.1, GraphPad Software Corporation, Santiago, CA, USA). Additional multivariate analyses were conducted using R software (ver.4.2.2, R Foundation for Statistical Computing, Vienna, Austria).

Prior to parametric testing, the normality of continuous data distribution was assessed using the Shapiro–Wilk test, and the homogeneity of variances was evaluated using Levene’s test. For data following a normal distribution and exhibiting homogeneous variances, Student’s *t*-test was employed for comparisons between two groups. For comparisons among three or more groups, a one-way analysis of variance (ANOVA) was performed. Principal component analysis (PCA) was applied to the normalized transcriptomic and metabolomic datasets to visualize sample clustering and detect potential outliers. Hierarchical cluster analysis (HCA) was performed using the Complex-Heatmap package in R to illustrate metabolite accumulation patterns across different samples. Prior to PCA and HCA, all metabolite data were subjected to unit variance (UV) scaling for standardization.

All results are presented as mean ± standard deviation (SD) from at least three independent biological replicates. Statistical significance was defined as *p* < 0.05 (*) and *p* < 0.01 (**); comparisons without asterisks indicate no statistical significance (*p* ≥ 0.05).

## 3. Results

### 3.1. SA Promotes Seed Germination Under High-Temperature Stress

**Figure 1:**
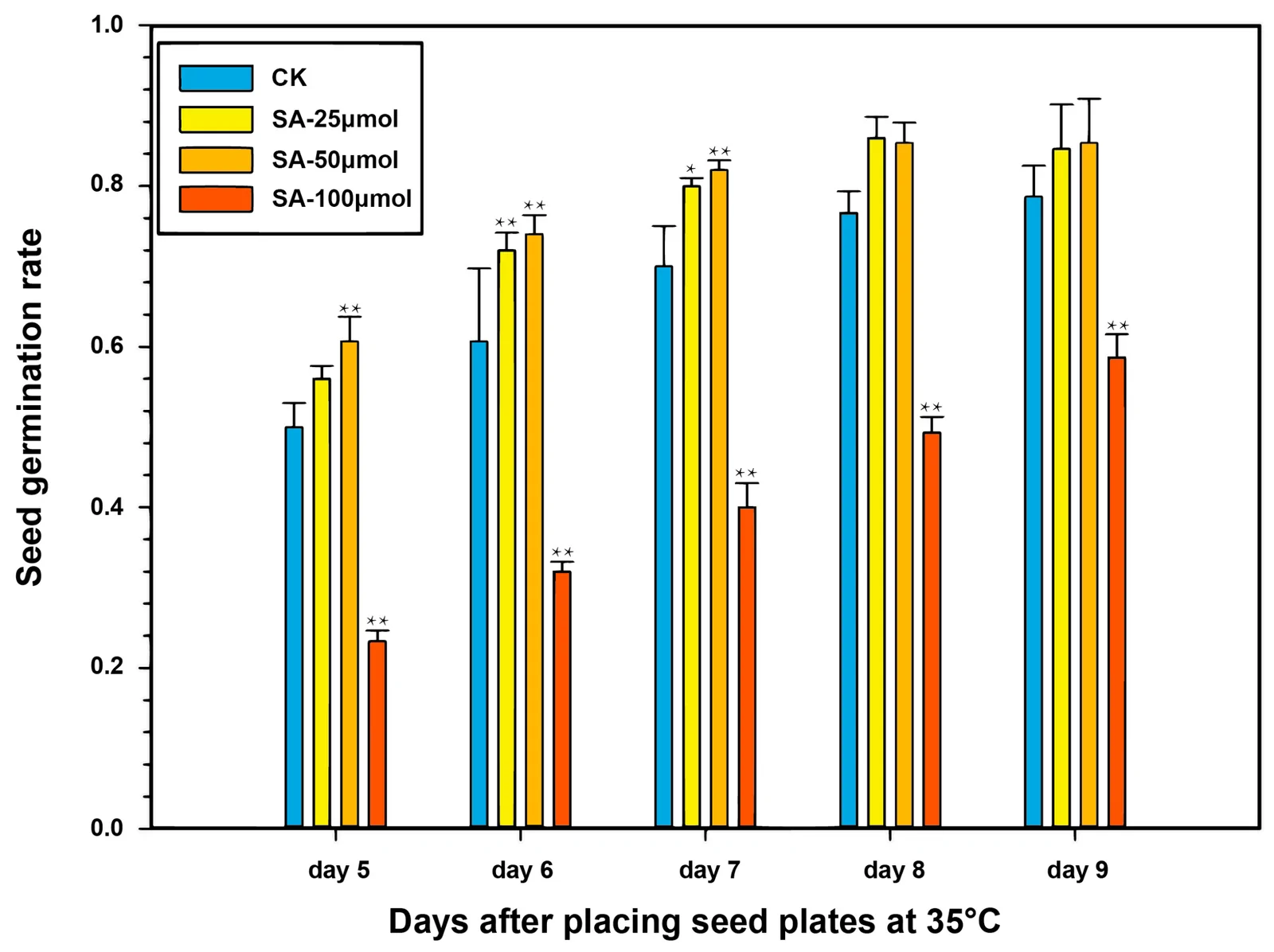
Seed germination rate of N7 under high-temperature stress in the presence of SA. The *X*-axis represents days after the plates containing seeds were placed at 35 °C. The *Y*-axis represents seed germination rate. * *p* < 0.05; ** *p* < 0.01. N = 3. Bars = means ± standard error of the mean.

To determine whether SA can alleviate high-temperature damage during the critical germination stage of *L. barbarum*, and to identify an effective concentration range for subsequent experiments, we evaluated seed germination under controlled high temperature conditions (35 °C) with varying SA concentrations. First, the influence of SA on seed germination under high temperature stress was determined by using N7. The experiment was divided into four groups: control, 25 μmol, 50 μmol, and 100 μmol SA groups. The seeds of N7 treated in the four groups were placed at 35 °C, and the germination conditions were observed. The seed germination rate was determined from the 5th day after transfer to 35 °C. As shown in Figure 1, the seed germination rate increased gradually from the 5th day, while the seed germination rates of 25 μmol/L and 50 μmol/L SA treatment groups were significantly higher than that of the control group, which increased by approximately 15% to 30%. The seed germination rate of the 100 μmol/L SA treatment group was significantly inhibited.

In summary, these results confirmed that SA at appropriate concentrations (25–50 μmol/L) effectively enhances seed germination under high temperature stress, whereas excessive SA (100 μmol/L) exhibited inhibitory effects. This concentration-dependent response informed the selection of SA concentration for the following field experiments. Having established the thermoprotective effect of SA at the germination stage, we next investigated whether this beneficial effect translates to improved yield and quality under field conditions during the warm growing season.

### 3.2. SA Treatment Enhances Fruit Yield and Mitigates Leaf Drop Under Field Conditions

Based on the thermoprotective effect of SA observed in the germination assays, we next examined whether SA application could improve agronomic performance under field conditions. Specifically, to study whether SA treatment enhances fruit yield and reduces leaf drop, a symptom exacerbated by high temperatures during the growing season in field, the field experiment was conducted during a hottest period during summer, providing natural heat stress conditions. The N7 shrub at the same age and growth condition were selected and divided into two groups. One was the control group (untreated plants, denoted as CK in Table 1 and figures), which was sprayed with leaf fertilizer, and the other was the experimental group (SA treated plants, denoted as SA in Table 1 and figures), which was sprayed with leaf fertilizer and SA. The statistical results of N7 yield were used as an example and are listed in Table 1. The statistical results showed that spraying SA increased the fresh fruit weight by 22%, the dried fruit weight by 22%, the seed number by 20%, and the seed weight by 22%, while reducing the fallen leaves by 32%, the weight of 100 fresh fruits by 13%, the weight of 100 dried fruits by 3%, and the ratio of fresh to dried fruit by 10%. In conclusion, the use of SA can significantly improve fallen leaf problem caused by global warming, and increase the production of N7. Simultaneously, the application of SA reduces the fruit weight of N7, especially in fresh fruit, which indicates that the water content has decreased but the organic matter ratio has increased in the fruits.

Taken together, these results demonstrated that SA treatment significantly improves multiple yield-related parameters, with a particularly notable reduction in leaf drop (32% decrease), which is consistent with the thermoprotective function of SA. However, yield enhancement alone does not guarantee commercial value; fruit nutritional quality is equally critical. We therefore proceeded to evaluate the impact of SA treatment on key fruit quality parameters.

### 3.3. SA Improves Fruit Nutritional Quality by Increasing Flavonoids and Soluble Sugars

**Figure 2:**
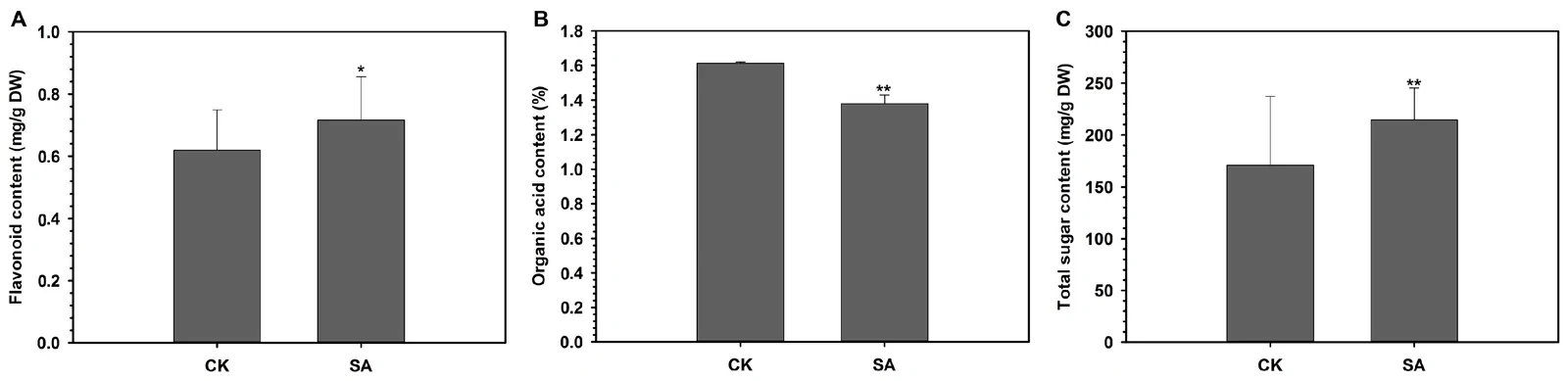
Effect of SA on the goji fruit composition of N7. (**A**) Flavonoid content; (**B**) organic acid; (**C**) total sugar content. CK: the control group (untreated plants), was sprayed with leaf fertilizer; SA: the experimental group (SA treated plants), was sprayed with leaf fertilizer and SA (0.05% (*w*/*v*)). The fruits were red goji fruits, which were collected 7 days after treatment. * *p* < 0.05; ** *p* < 0.01. N = 3. Bars = means ± standard error of the mean.

Given the observed yield improvement, we next asked: Does SA treatment also affect the nutritional quality of goji fruits, particularly the contents of flavonoids, soluble sugars, and organic acids, key determinants of commercial value and organoleptic properties. Next, the nutritional composition of N7 fruits with or without SA spraying was analyzed, as shown in Figure 2. The flavonoid content is shown in Figure 2A, the organic acid content is shown in Figure 2B, and the total sugar content is shown in Figure 2C. As the results show, the flavonoid content in N7 fruits increased by 14%, the organic acid content decreased by about 16%, and the total sugar content increased by 30% after SA addition under the same growth condition. This suggests that SA increases the nutritional value and sweetness but decreases the sour taste of N7 fruits.

These biochemical analyses revealed that SA not only boosts yield but also enhances the accumulation of nutritionally valuable compounds (flavonoids and total sugars) while reducing organic acid content, thereby improving the overall flavor and nutritional profile. To understand the molecular mechanisms driving these improvements at both transcriptional and metabolic levels, we subsequently performed transcriptomic and metabolomic profiling of fruits from control and SA-treated plants.

### 3.4. Transcriptome Analysis of N7 Fruit with SA Treatment

To elucidate the molecular basis underlying the SA-induced improvements in yield and fruit quality, we performed RNA-sequencing (RNA-seq) to compare the global transcriptomic landscapes of control and SA-treated fruits at both green and red ripening stages. This analysis aimed to identify key regulatory genes and metabolic pathways activated by SA treatment that may explain the observed phenotypic changes.

**Figure 3:**
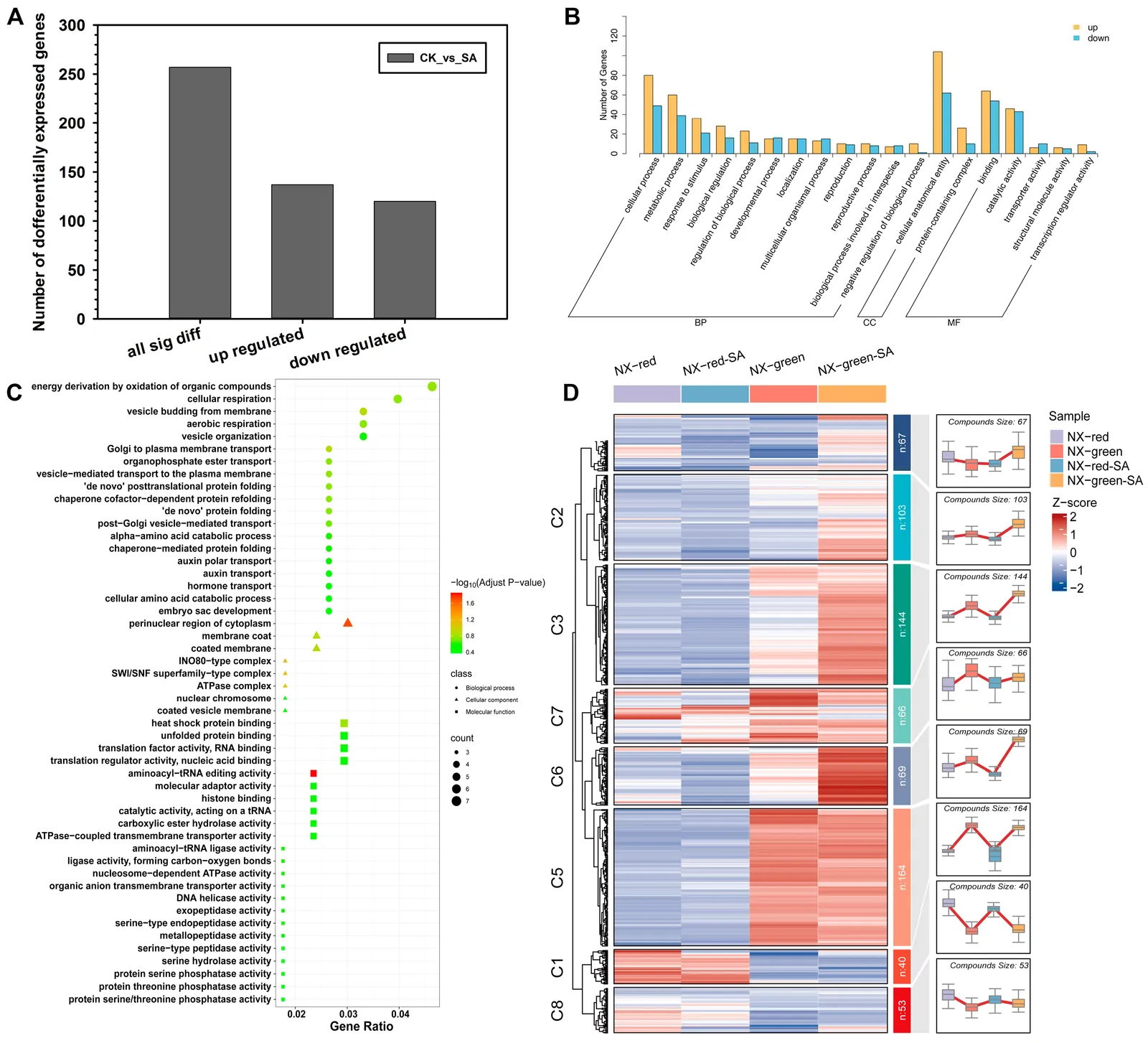
Transcriptome analysis of N7 fruit after SA treatment. (**A**) Number of DEGs in N7 fruit under SA treatment; (**B**) GO classification of DEGs; (**C**) GO enrichment analysis of top 50 DEGs between NX-red and NX-red-SA; (**D**) Z-score-normalized clustering heatmap and corresponding box-and-whisker plot. CK: the control group (untreated plants), was sprayed with leaf fertilizer; SA: the experimental group (SA treated plants), was sprayed with leaf fertilizer and SA (0.05% (*w*/*v*)). The fruits were red goji fruits, which were collected 7 days after treatment. The NX-red-SA and NX-green-SA samples correspond to red and green fruits, respectively, that were harvested 7 days after treatment with SA (0.05% (*w*/*v*)). In contrast, the NX-red and NX-green samples represent the untreated controls, consisting of red and green fruits collected at the same developmental stage without SA application.

Subsequently, transcriptome changes in N7 fruits after SA treatment were analyzed, and the results are shown in Figure 3. The fruit samples were collected from Ningxia-grown N7 plants and divided into four groups: red (NX-red), green (NX-green), and their SA-treated equivalents (NX-red-SA and NX-green-SA). The SA-treated groups were harvested seven days after foliar spraying, whereas the untreated groups were collected synchronously to serve as controls. As shown in Figure 3A, there were 257 genes differentially expressed in N7 fruits in response to SA spraying, of which 137 genes were upregulated and 120 were downregulated. The DEGs were analyzed according to gene ontology (GO) classification and are shown in Figure 3B. GO classification was divided into three categories: molecular function, biological process, and cellular component. The most regulated genes were classified into cellular anatomical entities, cellular processes, metabolic processes, binding, and catalytic activity processes.

In addition, GO enrichment analysis of top 50 DEGs between NX-red and NX-SA-red is shown in Figure 3C. The enriched terms were categorized into three main ontologies: Biological process (BP), cellular component (CC), and molecular function (MF). The *x*-axis represents the gene ratio, the size of each bubble corresponds to the number of enriched genes, with larger bubbles indicating more genes assigned to the term. The color gradient indicates the statistical significance level, represented as −log_10_(adjusted *p*-value), where red denotes greater significance. All adjusted *p*-values were computed using the Benjamini–Hochberg method to correct for multiple testing. Terms with adjusted *p* < 0.05 were considered significantly enriched. The figure simultaneously presents enrichment strength, gene count, and statistical significance, providing a comprehensive overview of the functional categories affected by SA treatment. In the biological process category, the DEGs were mainly enriched in energy derivation by the oxidation of organic compounds and cellular respiration. In the cellular component category, DEGs were first enriched in the perinuclear region of the cytoplasm and coated membrane. In the molecular function category, DEGs were first enriched in heat shock protein binding and unfolded protein binding processes. In all, the most significantly enriched terms are energy derivation by oxidation of organic compounds and perinuclear region of cytoplasm.

The Z-score-normalized clustering heatmap and corresponding box-and-whisker plot in Figure 3D clearly illustrates the differences in expression patterns of molecules across the four sample groups (NX-red, NX-red-SA, NX-green, NX-green-SA). All molecules were classified into eight expression clusters (C1-C8), with different clusters exhibiting distinct response characteristics to tissue type (red/green) and SA treatment. Among these, C5 (*n* = 164) is the largest cluster; its molecules are lowly expressed under red conditions and significantly highly expressed under green conditions, with expression changes unaffected by SA treatment. In contrast, molecules in clusters C2 (*n* = 103) and C3 (*n* = 144) exhibit a synergistic response to both conditions and treatment, reaching peak expression levels under green conditions following SA treatment. Molecules in modules C1 (*n* = 45) and C8 (*n* = 53) exhibited the opposite trend, with high expression under red conditions and suppressed expression under green conditions; the remaining modules (C6, C7, etc.) displayed transitional expression patterns intermediate between the aforementioned types. The box plots on the right further validate the overall expression trends of each module, visually presenting the average expression levels and variability of molecules within each module across different sample groups. Collectively, they reveal the differences in how various molecular groups respond to red/green conditions and SA treatment, providing a clear basis for classifying patterns in subsequent functional analyses.

### 3.5. Analysis of Regulated Genes in N7 Fruit in Response to SA Application

To further characterize the most responsive genes to SA treatment, we examined the top 20 upregulated and downregulated DEGs in N7 fruits. This analysis was designed to identify candidate genes that may play key roles in SA-mediated thermotolerance and quality improvement.

**Figure 4:**
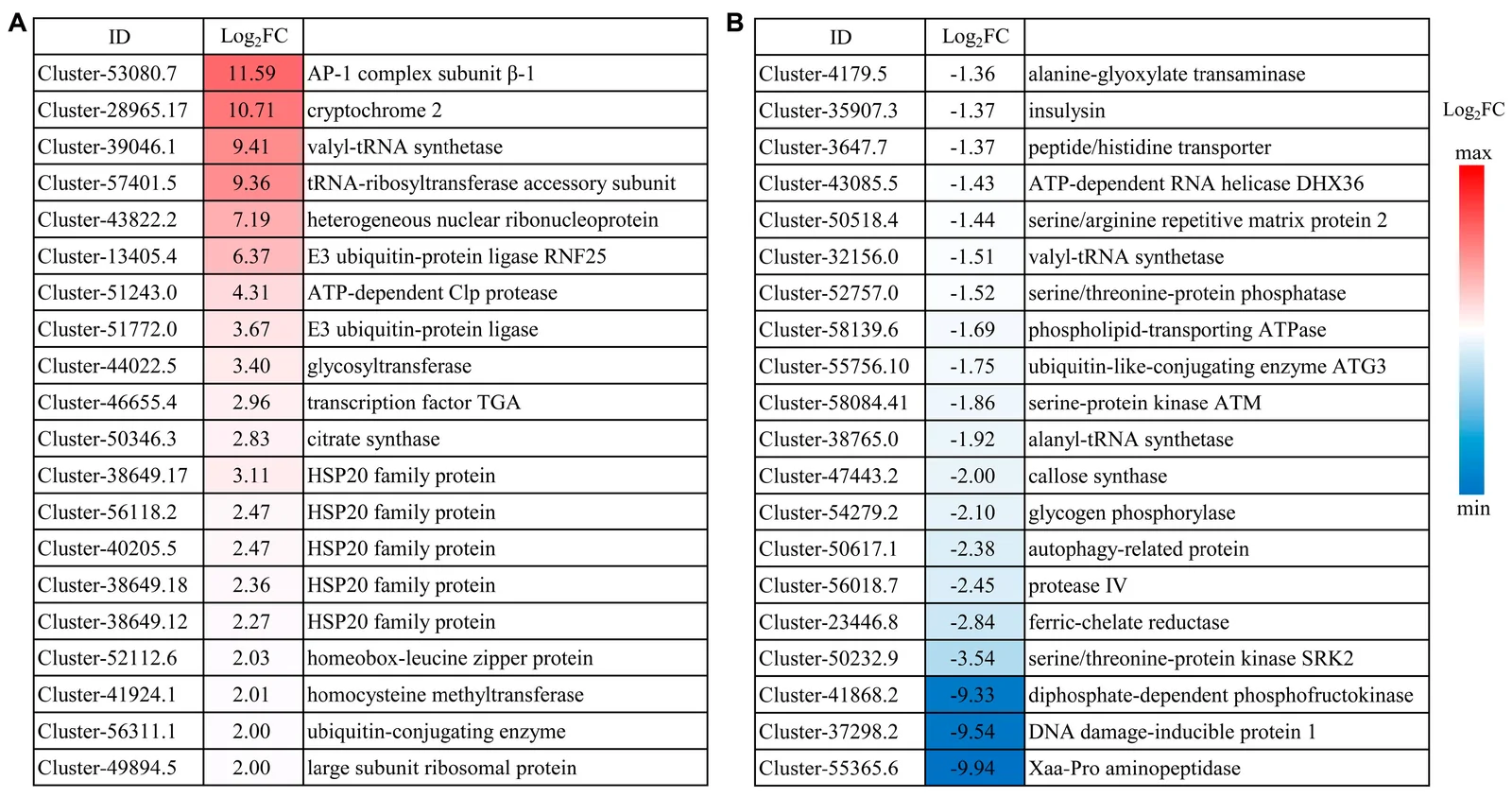
Analysis of DEGs in N7 fruit in response to SA treatment. (**A**) Genes that were upregulated in response to SA application in N7 fruit; (**B**) genes that were downregulated in response to SA application in N7 fruit.

The regulation levels of the top 20 upregulated and downregulated genes in N7 fruits in response to SA application under high temperature were analyzed and are shown in Figure 4. The top 20 upregulated genes are shown in Figure 4A and the top 20 downregulated genes are shown in Figure 4B. The top upregulated genes mainly included AP-1 complex subunit *β*-1, cryptic valyl-tRNA synthetase, tRNA ribosyltransferase accessory subunit, heterogeneous nuclear ribonucleoprotein, and the HSP20 family protein. The top downregulated genes included Xaa-pro aminopeptidase, DNA damage-inducible protein, diphosphate-dependent phosphofructokinase, serine/threonine protein kinase SRK2, ferric-chelate reductase, autophagy-related protein, and glycogen phosphorylase.

Overall, the transcriptomic analysis indicated that SA induces substantial reprogramming of gene expression, particularly affecting genes involved in energy metabolism, cellular respiration, and heat shock protein binding. Among the most notably upregulated genes were HSP20 family members and tRNA modification enzymes, suggesting enhanced protein homeostasis and translational regulation. These transcriptional changes provided the first layer of molecular evidence; however, whether these changes translate to actual metabolite accumulation remain to be determined. We therefore performed metabolomic profiling of the same samples.

### 3.6. Metabolomics Analyses of N7 Fruit in Response to SA

Since transcriptomic analysis revealed upregulation of genes involved in multiple biosynthetic pathways, we next performed UPLC-MS/MS-based metabolomic profiling to assess whether these transcriptional changes were accompanied by corresponding alterations in metabolite accumulation.

**Figure 5:**
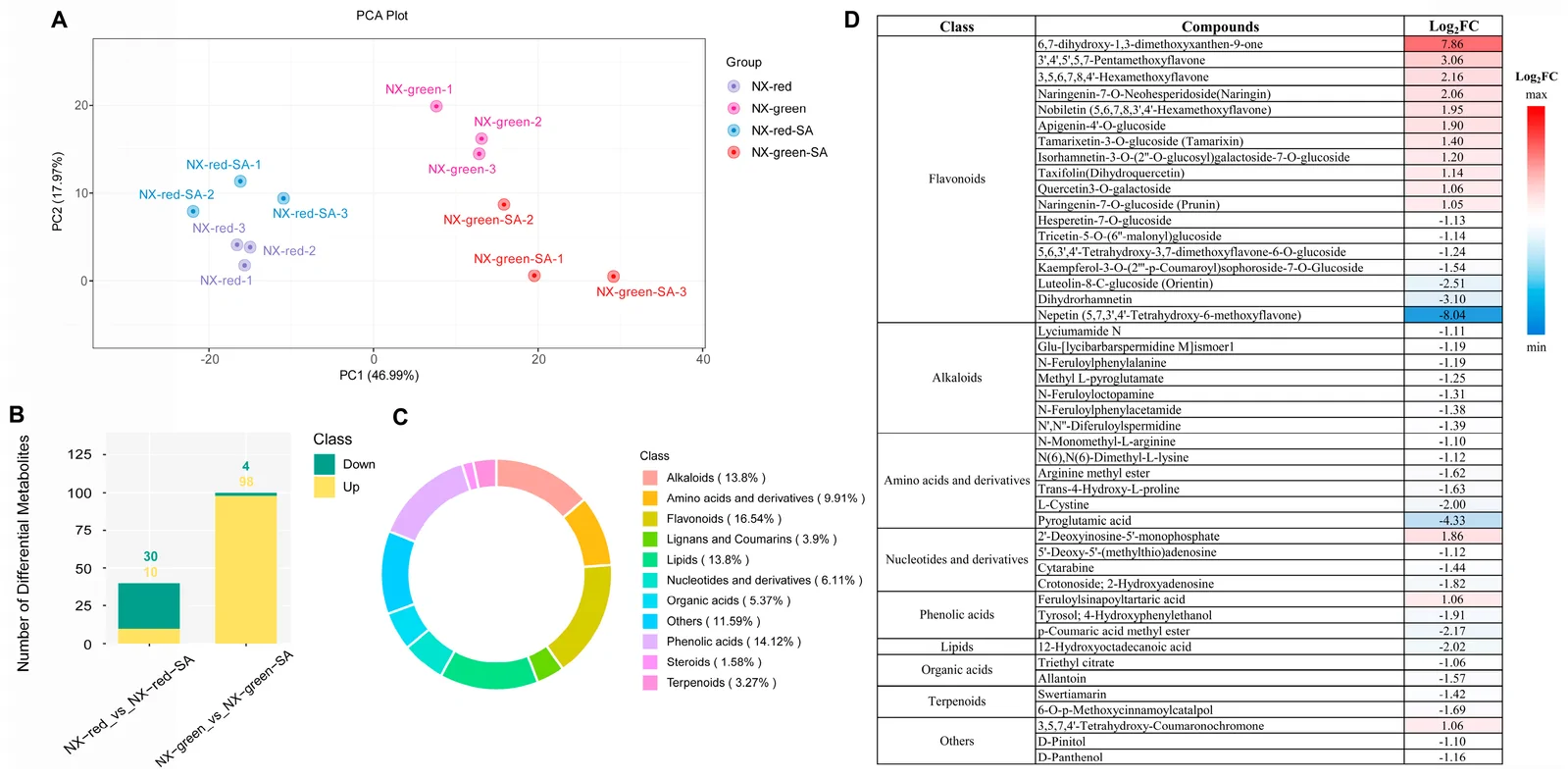
Metabolome analysis of N7 fruits in response to SA application. (**A**) PCA; (**B**) number of DAMs in N7 fruits under SA application; (**C**) class of DAMs in N7 fruit under SA application; (**D**) regulation pattern of DAMs in N7 fruit in response to SA.

The metabolomics results of this study in Figure 5 revealed metabolic differences between different sample groups through multivariate analysis: First, as shown in Figure 5A, the PCA score plot showed that PC1 and PC2 explained 46.99% and 17.97% of the total variance, respectively, for a combined total of 64.96%. The four sample groups: NX-red, NX-green, NX-red-SA, and NX-green-SA, exhibited a clear trend of intergroup separation. Specifically, red and green fruit samples were significantly separated along the PC1 axis, while SA-treated samples also showed a certain degree of distinction from untreated samples. This indicates that both fruit type and SA treatment exerted significant effects on the sample metabolome, and that intra-group sample reproducibility was good. Statistical analysis of differentially expressed metabolites revealed that the NX-red vs. NX-red-SA comparison group identified a total of 30 differentially accumulated metabolites (DAMs), including 10 upregulated and 20 downregulated metabolites; whereas the number of DAMs in the NX-green vs. NX-green-SA group was significantly higher, totaling 103, with 99 upregulated and only 4 downregulated (Figure 5B). This indicates that SA treatment caused far greater metabolic disruption in green fruit than in red tissue, with upregulation being the predominant trend. Classification analysis of the differentially expressed metabolites revealed that these substances were primarily composed of flavonoids (16.54%), alkaloids (13.8%), lipids (13.8%), phenolic acids (14.12%), and amino acids and their derivatives (9.91%) (Figure 5C). Furthermore, the heatmap presented in Figure 5D was employed for cluster analysis of metabolites that exhibited significant differences. The results indicate that the majority of significantly upregulated metabolites were flavonoids, including 6,7-dihydroxy-1,3-dimethoxyanthrone-9-one, 3′,4′,5′,5,7-pentamethoxyflavone, naringenin, and nobiletin, among others. Conversely, the downregulated metabolites primarily consisted of nepetin, dihydrorhamnetin, pyroglutamic acid, L-cystine, *p*-coumaric acid methyl ester, 12-hydroxyoctadecanoic acid, as well as other alkaloids and organic acids. These findings indicate that SA treatment induces extensive metabolic reprogramming, particularly in green fruits, through the regulation of these key metabolic pathways.

Collectively, the metabolomic data confirmed that SA treatment induces extensive metabolic reprogramming, with flavonoid accumulation being the most prominent feature, particularly in green fruits. Notably, the higher number of DAMs in green fruits suggests that SA exerts its most profound metabolic effects during early fruit development. These findings corroborate the transcriptomic results and provide direct evidence that SA enhances the accumulation of bioactive flavonoids. To gain a holistic understanding of how transcriptional and metabolic changes are coordinated, we next performed integrated pathway-level analysis.

### 3.7. Conjoint Analyses of Transcriptomics and Metabolomics

Having obtained both transcriptomic and metabolomic datasets, we sought to integrate these two layers of information to identify the core metabolic pathways coordinately regulated by SA. This integrative analysis was designed to pinpoint the pathways that best explain the SA-induced improvements in fruit quality at the molecular level and to identify correlated gene–metabolite pairs that may represent key regulatory nodes.

**Figure 6:**
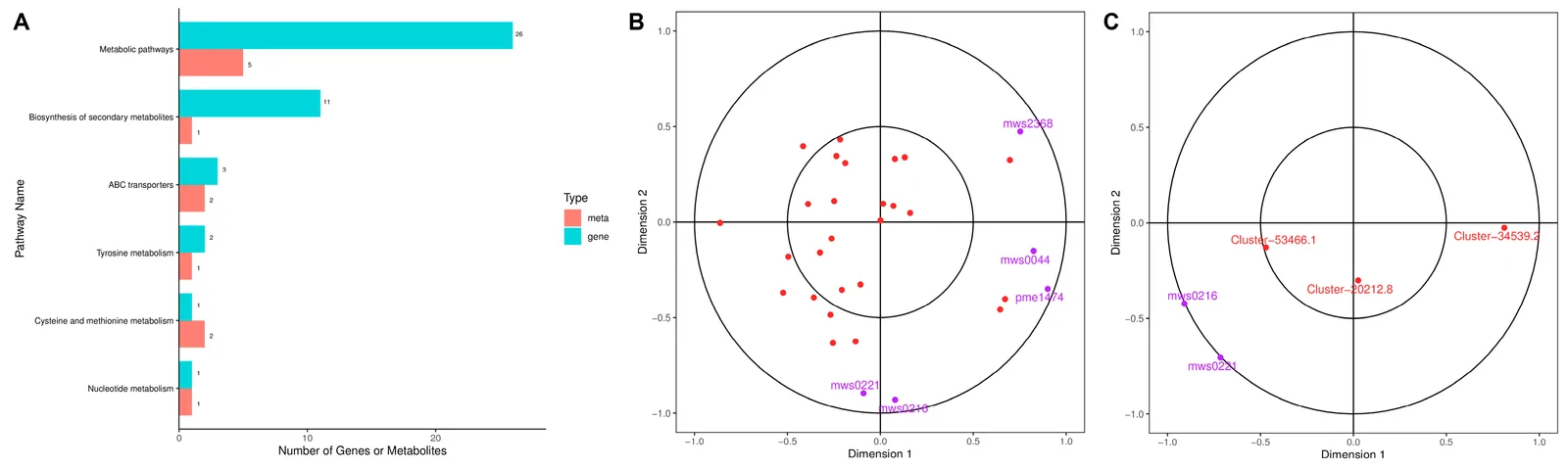
Conjoint analysis of transcriptome and metabolome data of N7 fruit in response to SA application. (**A**) KEGG enrichment of DEGs and DAMs in N7 fruit in response to SA application; (**B**) CCA plot of genes and metabolites that were enriched in the ko01100 pathway (metabolic pathway) in N7 fruit under SA treatment. pme1474: 5′-Deoxy-5′-(methylthio) adenosine; mws0216: Trans-4-Hydroxy-L-proline; mws0221: L-cystine; mws2368: Tyrosol; 4-Hydroxyphenylethanol; mws0044: Taxifolin (Dihydroquercetin); (**C**) CCA plot of genes and metabolites that were enriched in the ko02010 pathway (ABC transporters) in N7 fruit under SA treatment. mws0216: Trans-4-Hydroxy-L-proline; mws0221: L-Cystine. Metabolites are marked in purple, and genes are marked in red in CCA plot.

As shown in Figure 6, by combining the transcriptome and metabolome analyses, some metabolites and related genes were identified. The co-regulated DEGs and related metabolites were mainly included the ABC transporters, tyrosine metabolism, cysteine and methionine metabolism, and nucleotide metabolism pathways (Figure 6A). Moreover, a canonical correlation analysis (CCA) was conducted, in which some genes and metabolites were enriched in the metabolic pathway, as is shown in Figure 6B, and some metabolites and related genes were enriched in the ABC transporter pathway, as is shown in Figure 6C. In the CCA plot, four regions were distinguished using a cross. In the same region, the farther away from the origin, the closer they are to each other, and the higher the correlation between the points. The CCA plot in Figure 6B shows that metabolites pme1474 (5′-Deoxy-5′-methylthio-adenosine) and mws0044 (taxifolin, dihydroquercetin) have strong relationships, and the CCA plot in Figure 6C shows that metabolites mws0216 (trans-4-Hydroxy-L-proline) and mws0221 (L-cystine) were strongly correlated in N7 fruits in response to SA.

The integrated analysis revealed that ABC transporters, tyrosine metabolism, and sulfur-containing amino acid metabolism (cysteine and methionine) were the most prominently co-regulated pathways. The strong correlations between specific metabolites (e.g., 5′-deoxy-5′-methylthio-adenosine with taxifolin, and trans-4-hydroxy-L-proline with L-cystine) suggest coordinated regulation of these metabolic branches. To further dissect the specific pathways through which SA exerts its effects, we next examined the expression patterns of genes and accumulation of metabolites in three key metabolic pathways.

### 3.8. Coordinated Regulation of Lyciumspermidine, Flavonoid, and Betaine Biosynthesis Pathways by SA

Based on the integrated pathway enrichment analysis and the observed phenotypic improvements in fruit quality, we focused on three specific metabolic pathways that showed the most pronounced co-regulation: (1) Lyciumspermidine biosynthesis, (2) flavonoid biosynthesis, and (3) betaine biosynthesis. For each pathway, we examined the coordinated expression patterns of key enzyme-encoding genes and the accumulation of corresponding metabolites across developmental stages and treatment conditions.

**Figure 7:**
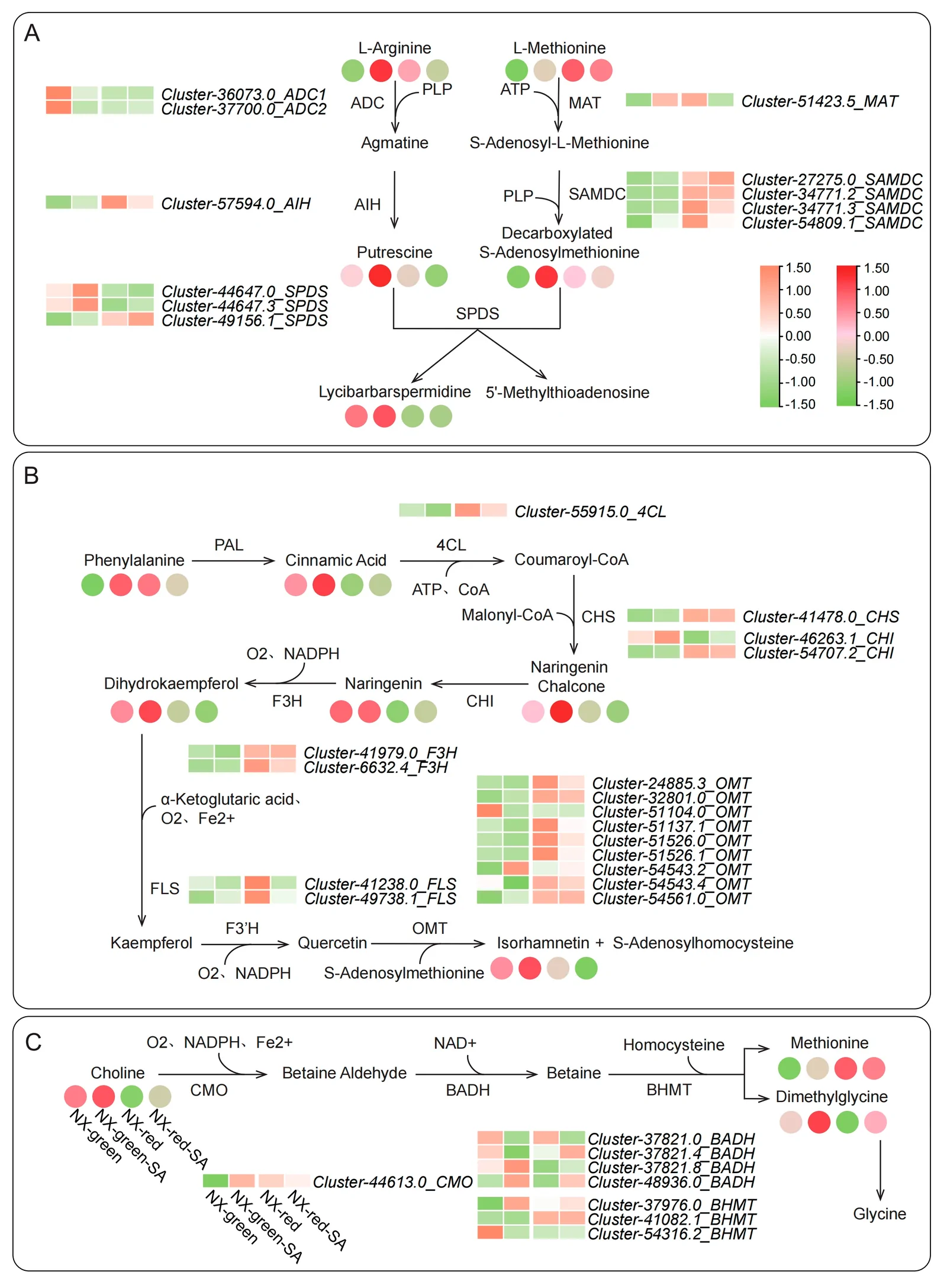
Expression pattern of DEGs and accumulated metabolites in key metabolic pathways. (**A**) Lyciumspermidine; (**B**) flavonoid; (**C**) betaine metabolic pathway. The heatmap displays log_2_ (fold change) expression levels, with red indicating upregulation and green indicating downregulation. ADC, arginine decarboxylase; AIH, agmatine iminohydrolase; MAT, methionine adenosyltransferase; SAMDC, S-adenosylmethionine decarboxylase; SPDS, spermidine synthase; PAL, phenylalanine ammonia lyase; 4CL, 4-coumarate CoA ligase; CHS, chalcone synthase; CHI, chalcone isomerase; F3H, flavanone 3-hydroxylase; FLS, flavonol synthase; OMT, O-methyltransferase; CMO, choline monooxygenase; BADH, betaine aldehyde dehydrogenase; BHMT, betaine homocysteine S-methyltransferase.

As shown in Figure 7, through a combination of pathway mapping and expression heatmaps, the changes in the expression and accumulation of core genes and metabolites across three key pathways—lyciumspermine synthesis, flavonoid synthesis, and betaine metabolism-in different sample groups (NX-red, NX-green, NX-red-SA, NX-green-SA). Specifically, in the lyciumspermidine synthesis pathway (Figure 7A), key enzyme genes such as *ADC*, *SAMDC*, and *SPDS*, as well as their downstream products putrescine and spermine, showed an overall upregulation trend in the NX-green and NX-green-SA fruits, suggesting that polyamine metabolism is significantly activated in green fruits. In the flavonoid biosynthesis pathway (Figure 7B), key enzyme genes such as *4CL*, *CHS*, *CHI*, *FLS*, and *OMT*, particularly *OMT* family genes, were significantly upregulated in the NX-green-SA samples. Corresponding intermediates such as naringin and quercetin also exhibited high accumulation in the green fruits, indicating that SA treatment further induces flavonoid biosynthesis in green fruits. In the betaine metabolic pathway (Figure 7C), the expression pattern of genes such as *CMO*, *BADH*, and *BHMT* showed that betaine synthesis-related genes were upregulated in both NX-green and NX-green-SA samples, while the metabolite choline accumulated at higher levels in green fruits, suggesting that SA treatment may participate in the stress response of green fruits by regulating betaine metabolism. Overall, key genes and metabolites in all three pathways exhibited distinct stage-specificity (higher activity during the green fruit stage) and SA-induced response characteristics, providing molecular level evidence for elucidating the differences in metabolic regulation of SA treatment in goji berries at different developmental stages.

In summary, the pathway-specific analysis revealed a consistent pattern: all three pathways, polyamine, flavonoid, and betaine biosynthesis, exhibited coordinated upregulation at both transcriptional and metabolic levels in response to SA treatment, with stronger effects observed at the green-fruit stage. These findings provide a molecular framework for understanding how SA simultaneously improves yield, nutritional quality (flavonoids and sugars), and stress tolerance (polyamines and betaine) in *L. barbarum*. The biological implications of these coordinated changes are further discussed later. Notably, while the present transcriptomic and metabolomic analyses were conducted under field conditions without controlled heat stress, the identified pathways-particularly polyamine and betaine biosynthesis, are well-established in the literature as heat stress-responsive pathways. The coordinated upregulation of these pathways by SA provides indirect molecular evidence supporting its thermoprotective function, although direct validation under controlled heat stress conditions remains an important direction for future research.

## 4. Discussion

Global warming has emerged as an escalating concern for global agricultural productivity. This study was designed to investigate the hypothesis that exogenous SA application can mitigate the negative impacts of summer hot weather on fruit quality in goji berry (N7), by modulating primary and secondary metabolic pathways. Specifically, we aimed to quantify the changes in sugars, organic acids, flavonoids, and alkaloids, and to elucidate the underlying molecular mechanisms through transcriptomic analysis. Our results demonstrate that SA treatment effectively reconfigures the metabolic landscape of N7 fruits under summer high temperatures, enhancing sugar and flavonoid accumulation, while suppressing organic acid and alkaloid biosynthesis. This trade-off ultimately improves the marketable yield and functional quality of the fruit.

### 4.1. Exogenous SA Reshapes Primary Metabolism, Favoring Sugar Accumulation over Organic Acid Synthesis

Following high-temperature treatment, the average concentrations of most organic acids in fruits increased, indicating that elevated temperatures promote organic acid accumulation [36]. This phenomenon may be attributed to enhanced respiratory metabolism and altered enzymatic activities under thermal stress. Research demonstrates that under heat stress, horticultural crops such as peppers and cucumbers sustain relatively high overall respiratory rates, with alternative respiration pathways playing a crucial role in enhancing thermotolerance. As a key intermediate in plant respiration, citric acid concentration initially declines due to accelerated consumption in energy metabolism but subsequently increases rapidly with rising temperature and prolonged stress duration, likely as a protective response. Elevated citric acid content contributes to improved heat stress resistance by stabilizing cellular pH and acting as an antioxidant [37,38]. Similarly, malic and tartaric acids may also accumulate, further influencing fruit taste and nutritional quality.

Furthermore, fruit quality is largely determined by sugar composition and concentration, primarily derived from photosynthetic assimilates produced in leaves. Under progressive thermal stress, total sugar and sucrose content in tomato fruits gradually decreases across various developmental stages following heat treatment during flowering and fruit set [39,40]. Such changes directly affect sweetness, flavor balance, and overall consumer acceptance.

Our data reveal a pivotal finding: SA application significantly increased the total sugar content in N7 fruits under hot weather, concurrently reducing the levels of most organic acids. This inverse relationship suggests an SA-driven metabolic redirection. High temperatures typically accelerate respiratory catabolism, which initially depletes organic acids like citric acid as intermediates in the TCA cycle, before a subsequent compensatory accumulation occurs as a stress-protective response. However, our results diverge from this general model; despite the hot weather, SA-treated fruits exhibited lower organic acid levels than untreated controls. We interpret this as evidence that SA does not merely alleviate stress but actively promotes the utilization of organic acid carbon skeletons as precursors for sugar and secondary metabolite biosynthesis. This is consistent with the role of SA in modulating source–sink relationships and phloem loading, as previously observed in tomatoes under heat–humidity stress. Critically, our transcriptomic data support this, showing SA-induced downregulation of diphosphate-dependent phosphofructokinase, a key glycolytic enzyme. Its downregulation likely slows the glycolytic flux, conserving carbon skeletons that are instead channeled into sugar accumulation, thereby directly improving the sweetness and flavor profile of the fruit-a primary quality attribute for consumers.

### 4.2. SA Specifically Enhances Flavonoid Biosynthesis While Suppressing Alkaloid Accumulation: A Functional Trade-Off

Secondary metabolites constitute the principal pharmacologically active constituents in many medicinal plants. For most plant species, secondary metabolite biosynthesis is often induced by environmental stimuli such as temperature extremes, UV radiation, or pathogen attack [41]. As an important traditional Chinese medicinal resource, goji berry conforms to this pattern. Flavonoids represent a class of polyphenolic compounds that enhance pigmentation, provide flavor characteristics, and mitigate oxidative stress in plants. Concurrently, they exhibit bioactive properties beneficial to human health, including anti-inflammatory, antioxidant, anti-obesity, anticancer, and antidiabetic effects, rendering them valuable ingredients in functional foods [3,4,5]. Alkaloids function not only as chemical defense compounds in plants but also as therapeutically active components in medicinal species, demonstrating anti-inflammatory, antibacterial, vasodilatory, and anticancer activities [42,43,44].

Previous reports suggest that SA can enhance plant thermotolerance through the modulation of antioxidant systems, stress-responsive gene expression, and photosynthetic efficiency; however, its effects on fruit yield and quality under realistic high-temperature scenarios remain insufficiently characterized. In this investigation, results demonstrated that exogenous SA application increased goji berry yield, seed content, flavonoid concentration, and total sugar levels while reducing organic acid and alkaloid accumulation in N7 fruits. These outcomes suggest that SA not only alleviates heat-induced physiological damage but also redirects metabolic fluxes toward sugar and flavonoid synthesis, thereby improving both marketable yield and functional quality of goji berry under thermal stress. Meanwhile, the application of SA upregulated key stress-responsive proteins, including the AP-1 complex subunit *β*-1, several E3 ubiquitin proteins, and various heat shock proteins, which are known to contribute to protein stabilization and stress adaptation. Concurrently, SA downregulated the expression of DNA damage-inducible protein, diphosphate-dependent phosphofructokinase-an enzyme involved in glycolytic regulation-and serine/threonine protein kinase SRK2, which plays a role in signal transduction under abiotic stress.

The most striking finding of this study is the opposing effect of SA on secondary metabolites, a significant increase in flavonoid content coupled with a marked decrease in alkaloid accumulation. While it is well-established that abiotic stresses, including heat, typically induce the biosynthesis of secondary metabolites as a defense mechanism [45], our results show that SA fine-tunes this response in a pathway-specific manner.

The upregulation of flavonoids under SA treatment aligns with their established role as potent ROS scavengers, crucial for mitigating heat-induced oxidative damage. This increase likely contributes to the enhanced antioxidant capacity and improved color characteristics of the treated fruits, directly enhancing their functional food value [46]. However, the concurrent suppression of alkaloids is counterintuitive, as they are also known to be induced under stress. We propose that this represents a strategic metabolic trade-off. Alkaloid biosynthesis is energetically expensive and their accumulation can sometimes be detrimental to fruit palatability due to bitterness. By downregulating alkaloid synthesis, the plant may be reallocating resources (e.g., nitrogen and carbon skeletons) toward the more urgently needed antioxidant defense system (flavonoids) and primary metabolites (sugars), which are essential for osmotic adjustment and energy storage under prolonged heat. This suggests that SA acts not as a general elicitor of all defense pathways, but as a sophisticated regulator that prioritizes specific protective mechanisms (flavonoids) over others (alkaloids), thereby optimizing both stress tolerance and fruit quality. This specific regulatory effect on different classes of secondary metabolites has not been extensively documented in goji berry and represents a key contribution of our work.

### 4.3. SA-Mediated Improvement in Yield, Taste, and Color Involves a Trade-Off Between Fruit Number and Size

Our physiological and transcriptomic data collectively demonstrate that SA application improves multiple agronomic traits of N7 fruits under summer hot weather, yet this improvement is accompanied by a distinct trade-off between yield components and individual fruit quality attributes.

SA treatment significantly increased the overall yield of N7 fruits while concurrently decreasing average fresh fruit size. This inverse relationship is not merely a passive consequence of reduced water content, as we initially hypothesized, but likely reflects an SA-induced shift in source–sink dynamics and dry matter partitioning. The upregulation of HSPs and E3 ubiquitin ligases observed in our transcriptomic data suggests that SA enhances protein homeostasis and reduces heat-induced proteotoxic stress at the cellular level, thereby promoting successful flowering and fruit set under otherwise suboptimal temperatures. However, the increased sink number (more fruits per plant) creates intensified competition for limited photosynthetic assimilates, resulting in reduced individual fruit expansion. This interpretation is supported by the downregulation of serine/threonine protein kinase SRK2, a negative regulator of ABA signaling, which may attenuate stress-induced growth inhibition and permit continued reproductive development, but at the cost of allocating fewer resources to each developing fruit. Unlike previous studies that reported SA-induced fruit size increase in tomato under optimal conditions, our findings suggest that under heat stress, the yield-promoting effect of SA operates primarily through enhanced survival and set rate rather than through enhanced fruit expansion.

It is important to acknowledge that our current transcriptomic analysis was conducted under non-stress conditions to isolate the direct SA-regulated transcriptional network. While this approach successfully identified SA-responsive genes-including HSPs, E3 ubiquitin ligases, and SRK2-that are plausibly involved in thermotolerance, the direct molecular validation of SA-mediated heat protection requires future RNA-Seq profiling under actual heat-stress conditions. Nevertheless, the consistency between our transcriptomic candidates and established heat-response pathways provides a robust foundation for generating testable hypotheses regarding SA mode of action in goji berry under thermal stress.

Our findings suggest that SA application offers a pragmatic, low-cost strategy for stabilizing goji berry production under rising summer temperatures, particularly for markets that prioritize sugar content, flavonoid bioactivity, and visual appeal over large fruit size. However, for cultivars or production systems where fruit size commands a price premium, the yield–size trade-off identified here must be carefully managed. Future research should explore the synergistic potential of SA with osmoregulators (e.g., proline or glycine betaine) to mitigate fruit size reduction while preserving the beneficial effects on sugar and flavonoid accumulation, thereby offering a more comprehensive climate-resilient solution for goji berry cultivation.

## 5. Conclusions

In summary, we investigated the effects of SA on goji berry yield increasement, fruit quality improvement, and seed thermotolerance. Our findings revealed that SA treatment markedly improved goji seed thermotolerance by increasing the seed germination rate under high-temperature stress. The current transcriptomic data reflect the SA-regulated network under non-stress conditions, and that direct validation of the heat-tolerance mechanism requires further RNA-Seq analysis under heat stress. Specifically, SA increased goji berry yield by approximately 20%, while concurrently boosting sugar accumulation, flavonoid content, and several other key metabolites associated with health benefits under summer high temperature. In contrast, it led to a reduction in organic acid and alkaloid levels, which may positively influence fruit palatability and nutritional value. These results underscore the efficacy of SA in promoting goji berry resilience and overall crop performance under adverse conditions. These observations further confirm that SA serves as an effective regulator for promoting the productive performance and fruit quality of goji berry.

## Figures and Tables

**Table 1:** Effect of SA on the yield of N7.

Yield of N7	CK (Control)	SA (Treated)	Ratio (SA/CK)	Increase /Decrease	*p* Value
Fallen leaves (Average tree, g)	515.56 ± 91.02	350.56 ± 85.80	0.68	−0.32	**
Fresh fruit weight (Average tree, kg)	4.56 ± 0.24	5.56 ± 0.27	1.22	0.22	**
Dried fruit weight (Average tree, kg)	0.96 ± 0.05	1.17 ± 0.06	1.22	0.22	**
Fresh fruit weight of one hundred grains (g)	94.31 ± 4.70	82.40 ± 7.72	0.87	−0.13	*
Dried fruit weight of one hundred grains (g)	16.96 ± 1.61	16.45 ± 1.11	0.97	−0.03	/
Ratio (Fresh fruit/Dried fruit)	5.56 ± 0.32	5.01 ± 0.33	0.90	−0.10	/
Seed number of ten fruits	288.00 ± 23.46	345.11 ± 49.68	1.20	0.20	**
Seed weight of ten fruits (g)	0.20 ± 0.01	0.25 ± 0.01	1.22	0.22	**
Number of seeds in one gram	1427.78 ± 16.60	1437.89 ± 4.07	1.01	0.01	/
Weight of a thousand seeds (g)	0.74 ± 0.03	0.70 ± 0.01	0.95	−0.05	/

Note: “CK” is the control group (untreated plants), which was sprayed with leaf fertilizer, “SA” is the experimental group (SA treated plants), which was sprayed with leaf fertilizer and SA. The “Ratio (SA/CK)” is a relative fold-change index calculated as the mean value of the treated group (SA) divided by the mean value of the untreated control (CK). A ratio > 1.00 indicates a treatment-induced increase, while a ratio < 1.00 indicates a decrease relative to the control. * *p* < 0.05; ** *p* < 0.01.

## Data Availability

The original contributions presented in this study are included in the article. Further inquiries can be directed to the corresponding authors.
